# S2k-Leitlinie Diagnostik und Therapie der Endometriose – Anforderungen an die Pathologie

**DOI:** 10.1007/s00292-021-00978-x

**Published:** 2021-10-01

**Authors:** Lars-Christian Horn, Anne Kathrin Höhn, Stefanie Burghaus, Sebastian Daniel Schäfer, Uwe Andreas Ulrich, Dietmar Schmidt, Mitglieder der AWMF-Leitlinienkommission zur Erstellung der S2k-Leitlinie Endometriose

**Affiliations:** 1 Abteilung Mamma‑, Gynäko- & Perinatalpathologie, Institut für Pathologie, Universitätsklinikum Leipzig AöR, Liebigstraße 26, 04103 Leipzig, Deutschland; 2 Frauenklinik, Universitätsklinikum Erlangen, Erlangen, Deutschland; 3 Klinik für Frauenheilkunde und Geburtshilfe, Universitätsklinikum Münster, Münster, Deutschland; 4 Frauenklinik, Martin-Luther-Krankenhaus, Berlin, Deutschland; 5 Institut für Pathologie Trier, Trier, Deutschland

**Keywords:** Endometriose, Karzinom, Diagnostik, Histopathologie, Interdisziplinäre Leitlinie, Endometriosis, Carcinoma, Diagnostic histopathology, Workup, Recommendations

## Abstract

Die vorliegende Übersicht fasst die relevanten Aspekte der S2k-Leitlinie Endometriose zusammen. Die Empfehlungen umfassen die Aufarbeitung und Befunderhebung bei Biopsien und Resektaten, die bei der klinischen Diagnose einer Endometriose entnommen wurden. Die Leitlinie berücksichtigt neben praktischen Aspekten der Pathologie ebenso die klinischen Notwendigkeiten an die Histopathologie für eine optimale Diagnostik und Therapie der Patientinnen.

Basierend auf der in der pathologischen Literatur gebräuchlichsten Definition der Endometriose des Corpus uteri (Adenomyosis uteri) wurde diese in der Leitlinie definiert als der Nachweis des Endometrioseherdes im Myometrium in einem Abstand zur endomyometranen Grenze von einem mittelgroßen Gesichtsfeld (100fache Vergrößerung), was metrisch rund 2,5 mm entspricht. Bei Darmresektaten soll zum Status der Resektionsränder Stellung genommen werden.

Ebenso definiert werden Anforderungen im Kontext endometrioseassoziierter Karzinome (z. B. Hormonrezeptorbestimmungen, immunhistochemische Untersuchung auf Ausfall der DNA-Mismatch-Reparaturproteine).

## Abstract (en)

The present article summarises the recommendations for the handling, histopathological workup, diagnostics and reporting in surgical pathology of biopsies and resection specimens in patients with the clinical diagnosis of endometriosis. In addition to practical aspects of pathology, the guidelines also take into account the clinical requirements for histopathology for the optimal diagnosis and therapy of the patients.

Based on the definition of endometriosis of the corpus uteri (adenomyosis uteri) most commonly used in the pathological literature, this was defined in the guidelines as the detection of the endometriosis focus in the myometrium at a distance from the endomyometrial border of a medium-sized visual field (100× magnification), which in metric units corresponds to around 2.5 mm. In bowel resection specimens, the status of the resection margins had to be documented within the histopathological report.

Also mentioned are the requirements for the reporting of carcinomas associated with endometriosis, including the immunohistochemical evaluation of steroid hormone receptors and mismatch repair proteins.

Die interdisziplinäre Leitlinie für die Diagnostik und Therapie der Endometriose ist 2019 unter Schirmherrschaft der Arbeitsgemeinschaft der Wissenschaftlichen Medizinischen Fachgesellschaften e. V. (AWMF) erarbeitet worden. Am Anfang des Jahres 2020 wurde sie durch die beteiligten Fachgesellschaften und die AWMF konsentiert und im September 2020 publiziert (AWMF-Registernummer: 015-045).

Die aus dieser Leitlinie resultierenden Anforderungen für die Pathologie richten sich nach den Vorgaben internationaler Fachgesellschaften für die Endometriose sowie den diagnostischen und therapeutischen Anforderungen der Kliniker.

Im Text sind die jeweiligen konsensbasierten Statements, die z. T. auch den Qualitätsindikatoren der Leitlinie im Rahmen von Zertifizierungen entsprechen, rot hervorgehoben.

Hinsichtlich der Stärke der Empfehlung werden, in Analogie zu onkologischen Leitlinien der AWMF bzw. der Deutschen Krebsgesellschaft (DKG), 3 Empfehlungsgrade mit differenter Verbindlichkeit unterschieden, die sich auch in der Formulierung der Statements widerspiegeln.

In den nachstehenden Kapiteln sind die für die Pathologie relevanten Aspekte entsprechend des Textes der S2k-Leitlinie dargestellt.

Der vollständige Text kann als Langversion unter https://www.awmf.org/leitlinien/detail/ll/015-045.html abgerufen werden.

## Histologische Differenzialdiagnose der Endometriose

Aus histopathologischer Sicht besteht ein breites differenzialdiagnostisches Spektrum der Endometriose, das lokalisationsabhängig ist und nachfolgend kurz zusammengefasst werden soll.

**Figure 1:**
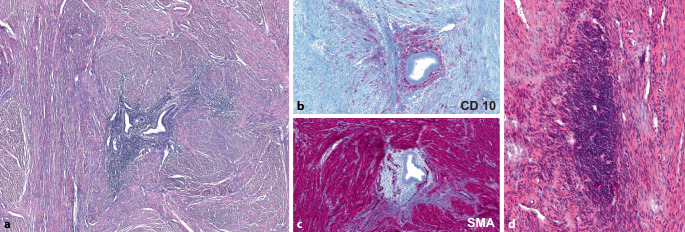

Die Diagnose der typischen Endometriose mit dem Nachweis endometrialer Drüsen und umgebendem Stroma bereitet in der Regel kein diagnostisches Problem (s. Abb. 1, 3 und 4; [8]). Frische und ältere Stromablutungen sowie Hämosiderophagen können vorkommen, seltener sind eine läsionale und periläsionale Fibrose sowie eine glattmuskuläre Metaplasie. Das endometriale Stroma ist immunhistochemisch positiv für CD10 (Abb. 1b), WT1 oder andere endometriale Stromamarker, die Histiozyten reagieren positiv für CD68. Insbesondere bei älteren Frauen und/oder nach vorangegangener medikamentöser Therapie der Endometriose können endometriale Drüsen fehlen oder sie sind nur fokal nachweisbar. Bei fehlendem Nachweis endometrialer Drüsen können (insbesondere bei peritonealen Biopsien) Stufenschnitte hilfreich sein. Lassen sich keine Drüsen und nur endometriales Stroma nachweisen, handelt es sich um eine *atrophe (syn. stromale) Endometriose* (Abb. 1e; [8, 9]).

Die wichtigsten Differenzialdiagnosen, insbesondere bei der extragenitalen Endometriose, sind die Endosalpingiose, die Endozervikose, die sog. Müllerianose, reaktive mesotheliale Proliferationen sowie maligne Tumoren [8, 17, 18, 33, 36] und in der Harnblase das nephrogene Adenom [33]. Empfehlenswert ist der Einsatz eines immunhistochemischen Panels, welches, je nach Befundkonstellation im HE-Schnitt, bei nichtneoplastischen Veränderungen die Marker CK7, CK20, ER, CD10 und ggf. CDX2 bzw. SATB2 umfassen sollte.

Lassen sich auch nach Anfertigung von Stufenschnitten und ggf. immunhistochemischen Zusatzuntersuchungen nur reaktive Veränderungen (wie Fibrose, Einblutungen, Histiozyten bzw. Hämosiderophagen) nachweisen, ist eine deskriptive Diagnose mit entsprechendem Kommentar zu empfehlen.

Lokalisationsbezogen wird die Morphologie der Endometriose im nachfolgenden kurz zusammengefasst.

Für den Terminus der Endometriose wird, insbesondere im angloamerikanischen Sprachraum, der Begriff der *Adenomyose* synonym gebraucht.

## Endometriose/Adenomyose des Corpus uteri

Die Endometriose/Adenomyose des Corpus uteri ist die häufigste Form der Endometriosis genitalis interna. Unter streng allgemeinpathologischen Gesichtspunkten stellt der Nachweis ektopen Endometriums im Myometrium ohne perifokale Veränderungen eine *Endometriosis uteri* (Abb. 1a,c,d) dar. Kommt es periläsional zu einer reaktiven (meist konzentrisch angeordneten) Proliferation des Myometriums, liegt eine *Adenomyosis uteri* (Abb. 1b) vor. Diese Unterscheidung ist jedoch diagnostisch und therapeutisch irrelevant. Im angloamerikanischen Schriftraum wird jeder Nachweis ektopen Endometriums im Myometrium als Adenomyosis bezeichnet und alle anderen Endometrioselokalisationen als Endometriose [52]. Insbesondere bei älteren Frauen kann auch nur eine *stromale Endometriose* vorliegen (s. Abschn. „Histologische Differenzialdiagnose der Endometriose“; Abb. 1e).

Für die histopathologische Diagnose der uterinen Endometriose gibt es keine allgemein akzeptierte Definition [9, 10, 13, 40].

Zumeist ist die Endometriosis/Adenomyosis uteri ein histologischer Zufallsbefund. Sie kann, insbesondere bei tief im Myometrium liegenden Herden, makroskopisch als Architekturstörung in Erscheinung treten [13]. Einzelne Studien berichten über eine Zunahme der klinischen Symptome mit größerer Tiefenlokalisation der Endometriose innerhalb des Myometriums [39].

Histologisch ist die Grenze zwischen Endo- und Myometrium nicht scharf, sondern undulierend [13].

**Figure 2:**
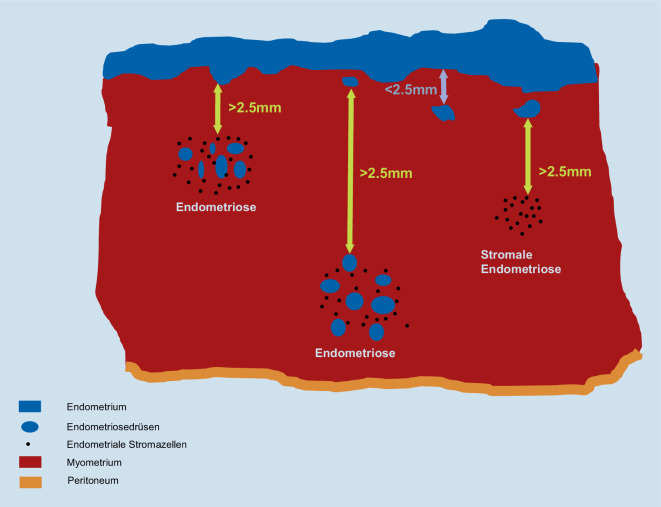

Die gebräuchlichste histopathologische Definition der uterinen Endometriose beinhaltet den Nachweis des Endometrioseherdes im Myometrium in einem Abstand zur endomyometranen Grenze von einem mittelgroßen Objektivfeld (100fache Vergrößerung). Metrisch entspricht dies ca. 2,5 mm (Abb. 2; [9, 10, 40]).

### Histopathologische Definition der Endometriose/Adenomyose des Corpus uteri

Histopathologisch ist die Endometriose des Corpus uteri (Adenomyosis uteri) definiert als der Nachweis des Endometrioseherdes im Myometrium in einem Abstand zur endomyometranen Grenze von einem mittelgroßen Objektivfeld (100fache Vergrößerung), was metrisch 2,5 mm entspricht.

Populationsbezogene Studien deuten darauf hin, dass Patientinnen mit einer uterinen Endometriose ein erhöhtes Risiko für ein Ovarial- bzw. Endometriumkarzinom des Endometriums aufweisen [31]. Dennoch ist das Risiko einer malignen Transformation einer Endometriosis uteri selbst als sehr gering einzustufen [16, 30]. Auch hier überwiegt das endometrioide Adenokarzinom; das klarzellige bzw. seröse Adenokarzinom ex Endometriosis uteri sind selten [13, 16, 30].

Das Einwachsen eines im Endometrium entstandenen Karzinoms in eine vorbestehende Endometriose kann ein in der Endometriosis uteri entstandenes Karzinom vortäuschen. Folgende, bereits in der S3-Leitlinie Endometriumkarzinom fixierten Befunde favorisieren die Diagnose der Mitbeteiligung einer Endometriose durch ein im Endometrium entstandenes Karzinom [1, 2, 20]:

- Nachweis benigner endometrialer Drüsen in unmittelbarer Nachbarschaft von Tumordrüsen,
- Nachweis benigner Drüsen zwischen den Tumordrüsen,
- fehlende peritumorale Desmoplasie,
- fehlende peritumorale Entzündung,
- in der kleinen Vergrößerung runde Außenkontur der Läsion mit scharfer Begrenzung zum umliegenden Myometrium.

## Bauchwandendometriose

**Figure 3:**
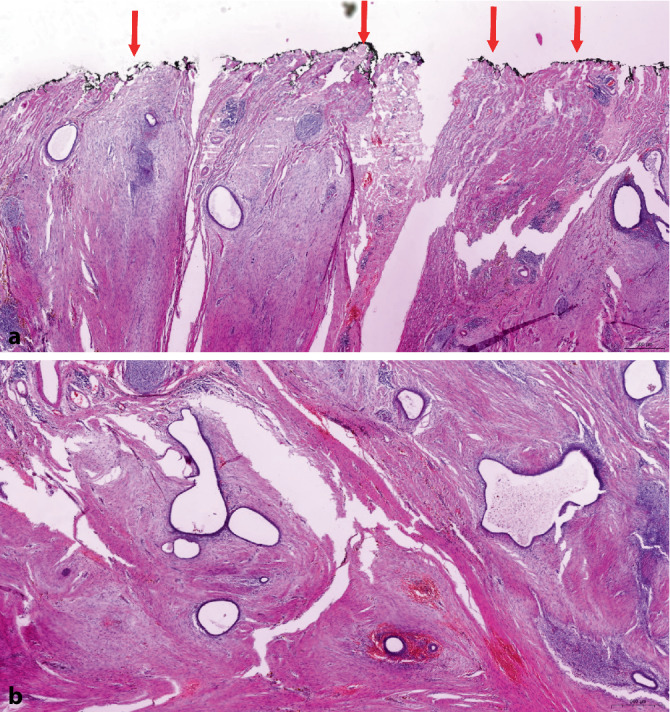

Die Bauchwandendometriose als häufigste Form der Weichteilendometriose stellt pathomorphologisch im Allgemeinen kein Problem dar. Dabei handelt es sich in den meisten Fällen um eine sog. *traumatische Endometriose*, bei er im Rahmen einer vorangegangenen Sectio caesaria Endometrium beim Eingriff in die Bauchdecke disloziert wurde (Abb. 3). In Abhängigkeit von der Hormonlage (z. B. Resektion im Rahmen einer Resectio) kann es zu sekretorischen Veränderungen der Drüsen und zu einer Stromadezidualisierung kommen. In nahezu allen Fällen handelt es sich um eine unilokuläre Läsion [55]. Wenn möglich, sollte im histopathologischen Befundbericht dazu Stellung genommen werden, ob der Befund vollständig reseziert wurde oder nicht. Dazu ist es zweckmäßig, den Resektionsrand farbig zu markieren (Abb. 3a). Eine maligne Entartung ist selten (47 Fälle bis 2017 beschrieben; [42]). Prädiktive Faktoren bezüglich der malignen Entartung sind nicht bekannt. Bei den Malignomen überwiegt mit rund zwei Dritteln das klarzellige gegenüber dem endometrioiden Karzinom. Ungünstige Prognosefaktoren sind eine klarzellige Histologie und beim endometrioiden Karzinom eine Tumorgröße von ≥ 8 cm [14, 42].

## Peritoneale Endometriose

**Figure 4:**
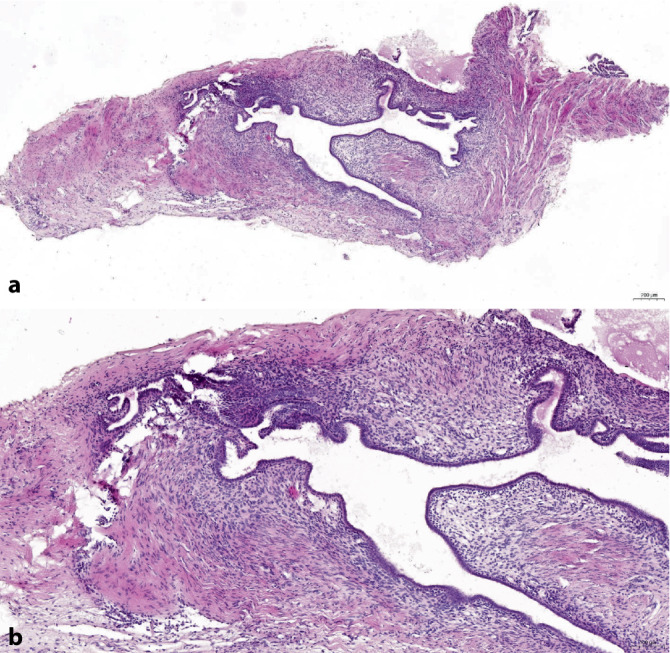

Die Endometriosis peritonealis (Abb. 4) ist häufig mit weiteren Endometrioselokalisationen assoziiert. In einem Teil der Fälle können sekundäre Veränderungen, wie Einblutungen, zystische Transformation oder umgebende entzündliche bzw. narbige Veränderungen dominieren. Psammoide Kalzifikationen können vorkommen und dann Probleme bei der Abgrenzung zur Endosalpingiose bzw. Implantaten seröser Borderlinetumoren führen. Hier sind immunhistochemische Untersuchungen hilfreich.

## Endometriose des Harntraktes

### Harnblase

Die Endometriose der Harnblase ist die häufigste Form der Endometriose des Harntraktes. Sie kann isoliert auftreten, ist jedoch meist mit einer tief infiltrierenden Endometriose bzw. einer Endometriosis genitalis assoziiert [6, 29, 32]. Innerhalb der Harnblase findet sie sich zumeist in der Detrusormuskulatur im Bereich des Trigonums vesicae und dem Apex der Harnblase [32, 51]. Die wichtigsten morphologischen Differenzialdiagnosen sind die Endozervikose (Nachweis von endozervikalem Zylinderepithel ausgekleideter Drüsen) und die sog. Müllerianose (Nachweis von Drüsen, die endozervikales Zylinderepithel, endometriales kubisches bzw. tuboendometrioides Epithel zeigen [17, 36]). Im Bereich des Harnblasendaches sind Reste des Urachus differenzialdiagnostisch zu bedenken [35, 48].

### Niere und Ureter

Die *Endometriose der Niere* ist extrem selten [11] und muss morphologisch von niereneigenen Läsionen wie Nierenrindenpapillomen, den seltenen nephrogenen Adenomen und nephrogenen Resten sowie Metastasen [56] abgegrenzt werden, was unter Einsatz der Immunhistochemie gelingt [54].

Die *Endometriose des Ureters* ist mit 14 % aller extra-pelvinen Endometrioselokalisationen selten [6, 29, 32] und zumeist mit einer tief infiltrierenden Endometriose assoziiert. Sie ist in der Majorität der Fälle unilateral und linksseitig sowie im distalen Drittel des Ureters lokalisiert [22, 32, 49]. Da in einem Teil der Fälle das periglanduläre endometrioide Stroma fehlen kann, ist die Abgrenzung gegenüber den von außen in das ureterale Meso oder die Ureterwand einwachsenden Adenokarzinomen wie lokal fortgeschrittenen Adenokarzinomen der Cervix uteri [53] oder Ovarial- bzw. seltener Rektumkarzinomen wichtig. Dabei können immunhistochemische Untersuchungen hilfreich sein.

In Abhängigkeit von der Lokalisation der Endometriose in der Ureterwand können 2 Formen der Ureterendometriose unterschieden werden:

1. die mit 20–30 % seltenere sog. intrinsische Ureterendometriose bei der die endometrialen Drüsen in der Uretermuskulatur bzw. Schleimhaut liegen und die
2. mit 70–80 % häufigere extrinsischen Form mit Nachweis von Endometriumdrüsen im periureteren lipofibrösen Gewebe; sog. Uretermeso) [6, 22, 32].

Obwohl die intrinsische Form aufgrund ihrer Ausdehnung nicht selten ein ausgedehnteres radikaleres Vorgehen impliziert [3, 6], liegen bezüglich der Rezidivhäufigkeit der extrinsischen und intrinsischen Form nur wenige Daten vor, sodass die Angabe der beiden o. g. Formen im histopathologischen Befundbericht fakultativ ist. Eine Stellungnahme zum Nachweis der Endometriose im Bereich der Resektionsränder (distal, proximal, zirkumferenziell) des Ureterteilresektates erscheint sinnvoll. Das Risiko einer malignen Transformation ist extrem niedrig [3].

## Darmendometriose

Bei Patientinnen mit ausgedehnter Endometriose breiten sich die Endometrioseherde im Weichgewebe und ggf. auch in andere Organe aus, was als (tief) infiltrierende bzw. invasive Endometriose bezeichnet wird. Hier ist morphologisch ein endometriales Stromasarkom auszuschließen, insbesondere beim fehlenden Nachweis endometrialer Drüsen.

Darmbeteiligung wird in rund 10 % der Patientinnen mit einer infiltrierenden Endometriose beobachtet [6]. Obwohl eine Ausdehnung der (Dick‑)Darmendometriose bis in die Submukosa nachweisbar ist [25], sind rektale Blutungen selten und endoskopische Biopsien oft nicht diagnostisch wegweisend [6].

**Figure 5:**
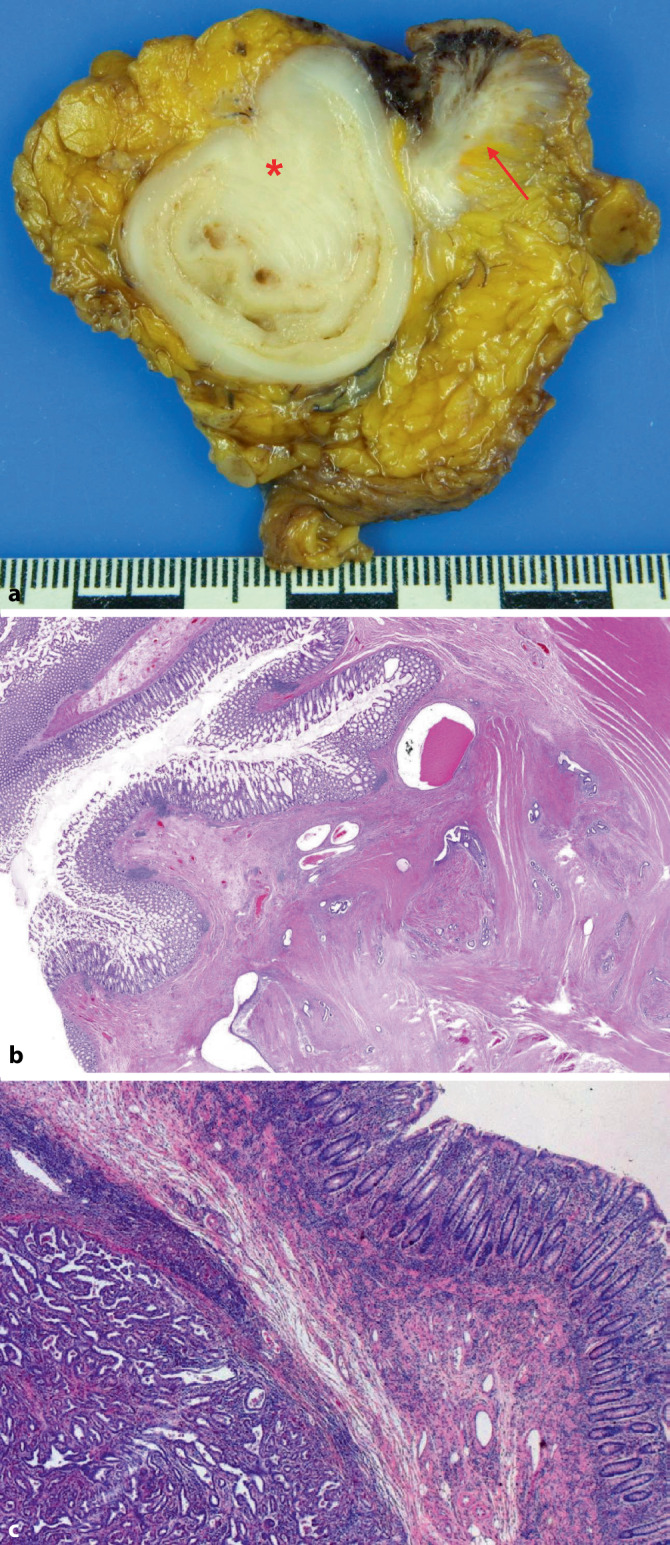

**Figure 6:**
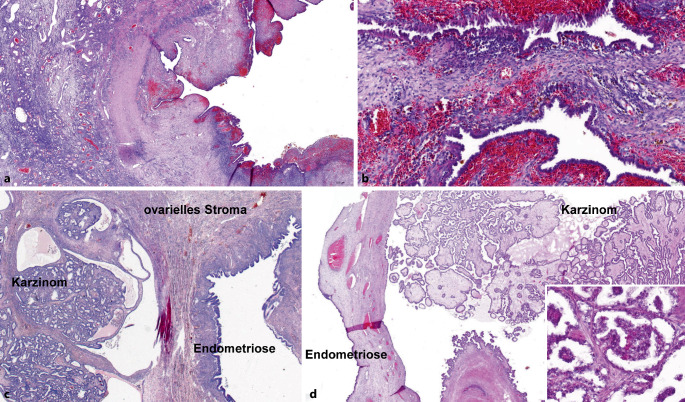

Da nur eine schlechte Korrelation zwischen der Ausdehnung der Darmendometriose und den klinischen Symptomen besteht, finden sich intestinale Symptome in der Regel bei Patientinnen, die periläsionale reaktive Veränderungen mit z. B. tumorähnlicher Verdickung der Darmwand aufweisen (Abb. 5; [6, 7, 21]). Bei diesen Patientinnen ist zumeist eine chirurgische Intervention indiziert, da mittels radiologischer Schnittbildverfahren ein Malignomausschluss nur unzureichend gelingt [43].

Makroskopisch sollte bei Darmresektaten die Angabe der Größe von (oft tumorähnlichen) Darmwandveränderungen [21] sowie deren Abstand zu den Resektionsrändern metrisch erfolgen. Da 50–60 % der (Rektum‑)Endometriosen multifokal und ein Drittel multizentrisch auftreten [25, 27], wird von einigen Autoren ein freier Resektionsrand von 2 cm empfohlen [27]. Zweckmäßig ist die separate Einbettung der Darmresektionsränder und die Entnahme repräsentativer Proben zur Beurteilung der Ausdehnung der Läsion in der Darmwand unter Berücksichtigung des Status und des Abstandes zum zirkumferenten (Weichgewebs‑)Resektionsrand.

Satellitenherde ohne eine Verdickung der Darmwand aufgrund sekundär reaktiver Veränderungen können in 50–60 % der Resektate beobachtet werden [25, 27] und sollten im Befundbericht erwähnt werden. Eine Beteiligung perikolischer Lymphknoten ist selten [21].

Eine Stellungnahme zum Nachweis der Endometriose im Bereich der Resektionsränder (distal, proximal, zirkumferenziell) soll erfolgen. In Zweifelsfällen können immunhistochemische Veränderungen zur Verifizierung der Diagnose sinnvoll sein.

### Resektionsrandbeurteilung bei Darmresektaten tief infiltrierender intestinaler Endometriosen

Bei Darmresektaten mit Endometriose soll zum Resektionsrandstatus im histopathologischen Befundbericht Stellung genommen werden.

Das Risiko einer malignen Transformation ist sehr gering. Zumeist handelt es sich um endometrioide Adenokarzinome; klarzellige und seröse (Abb. 5c) Karzinome sind selten [41]; endometriale Stromasarkome können vorkommen [28]. Bei serösen Karzinomen in der Darmwand (insbesondere rektosigmoidal) müssen Infiltrate eines high-grade serösen Tuben‑/Ovarial‑/Peritonealkarzinoms bzw. eines serösen Endometriumkarzinoms immunhistochemisch ausgeschlossen werden.

## Ovarielle Endometriose (Abb. 6)

Populationsbezogene Untersuchungen zeigen ein bis zu 4fach erhöhtes Risiko für ein Ovarialkarzinom bei Endometriosepatientinnen [31, 52]. Unter diesen überwiegt mit knapp zwei Drittel der endometrioide Subtyp, gefolgt vom klarzelligen Karzinom (20 %), seröse und muzinöse Karzinome sind selten [4, 37, 52]. Das seromuzinöse Zystadenom bzw. der seromuzinöse Borderlinetumor des Ovars [34] weisen ebenfalls eine Assoziation zu einer ovariellen Endometriose auf [26, 38, 52].

Aufgrund des erhöhten Malignomrisikos sollte eine adäquate Einbettung mit Entnahmen aus papillären Veränderungen und/oder Wandveränderungen der Endometriosezyste (Verdickungen, Fibrose, Konsistenzvermehrungen etc.) erfolgen. In Einzelfällen finden sich bei derartigen Untersuchungen Veränderungen im Sinne einer *atypischen Endometriose*. Als solche wurden in der Literatur 2 Formen definiert: zum einen komplexe atypische glanduläre Hyperplasie, die einer atypischen Hyperplasie des Endometriums ähnelt, zum anderen Atypien des auskleidenden Epithels einer Endometriosezyste, welches zusätzlich Zeichen einer unterschiedlich starken Proliferation und verschiedene Formen der Metaplasie (eosinophil, muzinös, ziliar, plattenepithelial, klarzellig) aufweisen kann. Diese Atypien können ganz umschrieben oder multifokal auftreten mit pleomorphen hyperchromatischen Kernen mit homogenisiertem Chromatin, die hobnailartig ins Lumen vorspringen oder kleine Papillen bilden können. Beschrieben wurden atypische mikropapilläre Proliferate mit aberranter p53-Expression [46].

Bei den meisten Endometriosezysten mit eosinophilen Atypien handelt es sich jedoch um reaktive Zellveränderungen mit entsprechend benignem klinischem Verlauf. Das gleichzeitige Auftreten von Adenokarzinomen und seromuzinösen Borderlinetumoren in Endometriosezysten mit den anderen Atypieformen weist jedoch darauf hin, dass es sich gelegentlich um präkanzeröse Veränderungen handelt. Die geschätzte Häufigkeit der malignen Transformation beträgt 0,5–1 % [38].

Untersuchungen der letzten Jahre legen nahe, dass sowohl endometrioide als auch klarzellige Ovarialkarzinome in Assoziation mit einer Endometriose eine bessere Prognose aufweisen als solche ohne [4, 5]. Daher wird empfohlen, bei diesen beiden histologischen Subtypen mittels einer adäquaten Einbettung eine assoziierte Endometriose des Ovars nachzuweisen bzw. auszuschließen und dieses Ergebnis im histopathologischen Befundbericht zu erwähnen.

### Ovarielle Endometriose und assoziierte Karzinome

Bei endometrioiden und klarzelligen Ovarialkarzinomen soll im histopathologischen Befundbericht erwähnt werden, ob eine assoziierte Endometriose vorliegt oder nicht.

In einzelnen Fällen kann ein seröses Ovarialkarzinom mit glandulärem Wachstumsmuster ein endometrioides Muster aufweisen, was differenzialdiagnostische Probleme bereiten kann. In diesem Kontext hilfreich kann die Tatsache sein, dass im Vergleich zu den weitaus häufigeren und oft bereits fortgeschrittenen high-grade serösen Ovarialkarzinomen, endometrioide Ovarialkarzinome meist im FIGO-Stadium I und II diagnostiziert werden [4]. High-grade seröse Ovarialkarzinome bei Patientinnen mit *BRCA*-Keimbahnmutation können bei Vorliegen des sog. SET-patterns (**s**olides, **e**ndometrioides und **t**ransitionalzelliges Wachstumsmuster) ein endometrioides Ovarialkarzinom vortäuschen [23, 45]. Daher können zur exakten histopathologischen Subtypisierung eines Ovarialkarzinoms mit endometrioider Morphologie und fortgeschrittenem Tumorstadium bzw. bekannter *BRCA*-Keimbahnmutation immunhistochemische Untersuchungen differenzialdiagnostisch hilfreich sein.

Die molekularen Veränderungen, die im Rahmen der malignen Transformation einer ovariellen Endometriose auftreten können, sind sehr heterogen [19, 52]. In Analogie zum Endometriumkarzinom zeigt ein kleiner Teil, insbesondere der bei jüngeren Frauen (bis 55. Lebensjahr), eine Assoziation zum Lynch-Syndrom [12, 15, 50]. Daher soll bei Patientinnen mit einem endometrioiden bzw. klarzelligen Ovarialkarzinom (mit und ohne assoziierte Endometriose) eine immunhistochemische Untersuchung der Mismatch-Reparaturproteine [44] in Analogie zum Vorgehen beim Endometriumkarzinom [2] erfolgen.

Beim endometrioiden Ovarialkarzinom weisen solche mit einer Steroidhormonrezeptorexpression eine bessere Prognose auf [47]. Auch kann diese ein mögliches therapeutisches Target darstellen, sodass in Analogie zum Endometriumkarzinom [2] eine immunhistochemische Bestimmung des Estrogen- und Progesteronrezeptorstatus erfolgen soll.

Klarzellige Karzinome sind in der Regel Hormonrezeptor-negativ.

### Hormonrezeptorbestimmung bei endometrioseassoziierten Karzinomen

Bei endometrioiden bzw. klarzelligen endometrioseassoziierten Karzinomen soll eine immunhistochemische Hormonrezeptorbestimmung erfolgen.

Ein Teil der Patientinnen mit endometrioseassoziierten Karzinomen, insbesondere solche im Ovar und jene unter 55 Jahren können eine Assoziation zum Lynch-Syndrom aufweisen [12, 15, 50]. Daher soll, in Analogie zum Vorgehen beim Endometriumkarzinom [2] eine immunhistochemische Untersuchung der Mismatch-Reparaturproteine erfolgen.

### Molekularpathologische Untersuchung des endometrioseassoziierten Karzinoms auf Mikrosatelliteninstabilität

Eine (molekular-)pathologische Untersuchung hinsichtlich Lynch-Syndroms im Tumorgewebe sollte bei jedem vor dem 60. Lebensjahr diagnostizierten endometrioseassoziierten Karzinom erfolgen.

Ein Teil der klarzelligen Ovarialkarzinome weist einen immunhistochemischen Verlust der Mismatch-Repair-Proteine auf, gelegentlich in Assoziation mit einer dichten lymphozytären Infiltration, was mit einer erhöhten PD-L1-Expression assoziiert ist [24].

## Endometriose und assoziierte Karzinome

Eine Endometriose kann mit einer Tumorerkrankung assoziiert sein. Insbesondere im Ovar treten endometrioide und klarzellige Karzinome in Assoziation mit einer Endometriose auf (s. Abschn. „Ovarielle Endometriose“).

Bei der Diagnose und der Typisierung eines endometrioseassoziierten Karzinoms soll die aktuelle WHO-Klassifikation zugrunde gelegt werden.

### Histologische Subtypisierung endometrioseassoziierter Karzinome

Der Terminologie und der morphologischen Diagnostik eines endometrioseassoziierten Karzinoms soll die jeweils gültige Auflage der WHO-Klassifikation zugrunde gelegt werden.

## Fazit für die Praxis

- Die Adenomyosis uteri ist definiert als der Nachweis von Endometrium innerhalb des Myometriums mit einem Abstand zur endomyometranen Grenze von einem mittelgroßen Gesichtsfeld (100fache Vergrößerung), was metrisch rund 2,5 mm entspricht.
- Bei Darmendometriose Angaben zum Status der Resektionsränder erforderlich.
- Bei in der HE-Färbung unklaren Befunden sind ggf. immunhistochemisch die Endosalpingiose, die Endozervikose, die sog. Müllerianose, reaktive mesotheliale Proliferationen sowie maligne Tumoren abzugrenzen.
- Bei endometrioseassoziierten Karzinomen sind die Hormonrezeptorbestimmung und die Analyse der DNA-Mismatch-Reparaturproteine sinnvoll.
- Extragenitale Endometriosen sind selten, hier sollten immer sekundäre Karzinominfiltrate ausgeschlossen werden.
- Im Gegensatz zu anderen Lokalisationen ist die ovarielle Endometriose mit einem erhöhten Karzinomrisiko assoziiert.
